# Public health risks of traditional zootherapeutic practices in Africa

**DOI:** 10.1016/j.onehlt.2025.101178

**Published:** 2025-08-20

**Authors:** Léa Fourchault, Abdallah Lamane, Nguinwa Mbakop Dimitri Romaric, Ganiyat Temidayo Saliu, Sophie Gryseels, Erik Verheyen, Farid Dahdouh-Guebas, Katharina Kreppel

**Affiliations:** a Operational Directorate Taxonomy and Phylogeny, Royal Belgian Institute of Natural Sciences, Brussels 1000, 29 Vautierstraat, Belgium; b Systems Ecology and Resource Management (SERM), Department of Organismal Biology, Université libre de Bruxelles, 50 Avenue Franklin D. Roosevelt, CPi 264/1 B-1050, Brussels, Belgium; c CentraleSupélec, Université Paris-Saclay, Gif-sur-Yvette 91192, 3 rue Joliot-Curie, France; d Department of Computer Engineering, Università degli Studi di Firenze, Firenze 50121, 4 Piazza San Marco, Italy; e Evolutionary Ecology Group (EVECO), Department of Biology, University of Antwerp, Antwerp 2000, 13 Prinsstraat, Belgium; f Ecology, Evolution & Genetics Research Group (bDIV), Department of Biology, Vrije Universiteit Brussel - VUB, Pleinlaan 2, B-1050, Brussels, Belgium; g Interfaculty Institute of Social-Ecological Transitions, Université Libre de Bruxelles - ULB, Av. F.D. Roosevelt 50, CPi 130/03, 1050 Brussels, Belgium; h Emerging Infectious Diseases Unit, Department of Public Health, Institute of Tropical Medicine, Antwerp, Nationalestraat 155, Antwerp, Belgium

**Keywords:** Ethnobiology, Ethnomedicine, Zoonosis, Spillover, Biosecurity, One health

## Abstract

Over five billion people globally, primarily rely on a plant- and animal-based pharmacopoeia. The topical application, injection, or ingestion of animal products – such as excreta, blood, or meat – likely facilitates the spillover of zoonotic pathogens. Certain practices use species known to be involved in the transmission of pathogens of public health relevance, such as filoviruses, poxviruses, or coronaviruses. This article aims to review zootherapeutic practices and the public health risk they entail for the African continent. We first conducted a systematic review of the scientific literature published until July 30th, 2023. We then created a categorical score reflecting the risk of zoonotic pathogen spillover for each recorded practice and compared this risk between regions and demographic groups. A total of 53 studies were included, half of which were published between 2020 and 2023. Nigerian practices were comparatively well documented. The mean risk score linked to practices occurring in eastern Africa was significantly higher than that of practices occurring in central Africa (*p* = 0.0008, p-adj = 0.008), western Africa (*p* = 2.5e-66, p-adj = 2.5e-65), and southern Africa (*p* = 2.55e-17, p-adj = 2.55e-16). Further, we found that children are overall at increased risk for pathogen spillover (*p* = 0.001, p-adj = 0.003), compared to adults. Where other forms of healthcare are inadequate or unavailable, traditional practices that balance cultural significance and public health risks should be encouraged. We suggest that local communities, traditional practitioners, researchers, and administrations should collaborate on (i) the elaboration of a pan-African collection of traditional practices, (ii) the regular monitoring of risks and benefits linked to such practices, including any comorbidities linked to hazardous preservatives, or the spillover of anti-microbial resistant pathogens; as well as (iii) the elaboration of culturally meaningful alternatives to the practices that entail higher risks than benefits.

## Highlights

• Traditional medical practices using animal tissue and excreta (zootherapeutic ethnomedicine) entail risks for public health.
• Literature on zootherapeutic ethnomedicine in Africa has doubled between 2020–2023 compared to the previous 30 years.
• Zootherapeutic practices are under-documented in all African countries, except Nigeria.
• There is a higher risk of zoonotic pathogen spillover in eastern and northern Africa compared to other African regions.
• Children are especially at risk of ethnomedicine-related pathogen spillover.

## Graphical abstract

**Figure:**
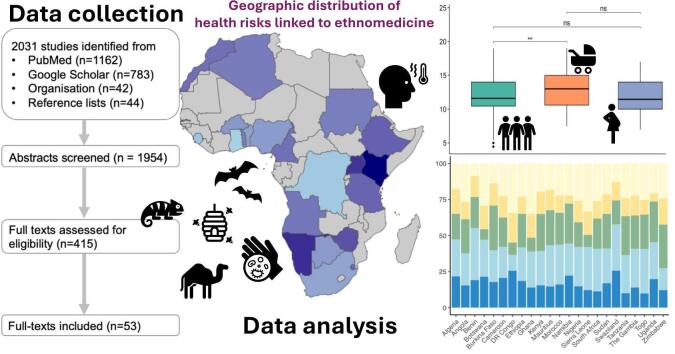

## 1 Introduction

Both wild and domestic animals host pathogens that can spill over to humans [[1], [2], [3], [4]], sometimes leading to major negative impacts on global public health and economies [5]. Examples include several HIV strains that originally spilled over from African primates [6], Mpox spilling over from African mammals (with the specific reservoir(s) still being investigated [7]), or *Brucella* bacteria from cattle, a predominant issue in northern and eastern Africa [8,9]. So far, hunting, and the butchering and consumption of wild and domestic animal meat have been investigated as major mechanisms for spillover, while other transmission routes, such as zootherapy, remain under-researched [[10], [11], [12], [13]].

Five billion people primarily rely on Traditional, Complementary, and Integrative Medicine (TCIM) for their healthcare and wellbeing, including about 80 % of the over one billion people inhabiting Africa [14]. Zootherapy*,* the use of animal *materia medica* (e.g.*,* fur, excreta, bones, blood), is an integral part of TCIM [15]. Zootherapeutic practices are a major source of exposure to animal products and the pathogens they may carry [16,17]. Each topical application, injection, inhalation, or ingestion of such animal products is therefore a potential mechanism for spillover [17,18]. Identifying zootherapeutic practices of higher epidemiological risk is thus crucial to develop sustainable alternatives that balance cultural significance and public health.

Reviews of zootherapeutic practices have previously focused on Asia, Latin America, and southern Europe [15,19,20]. A global review on the use of animals for health purposes has recently been published, with an emphasis on biodiversity and conservation rather than public health and restricted to mammals [16]. Other recent global reviews of zootherapeutic practices are restricted to specific ailments (e.g., analgesic properties of animals and plants [21] or anti-urolithiatic properties [22]), and do not examine public health impacts of such practices.

Our first aim was to analyse geographic and temporal trends in the recording of African zootherapeutic practices since 1990, to guide future research. Our second aim was to characterise geographic and demographic variations in the risk of zoonotic pathogen spillover, based on animal tissue types used as *materia medica*, methods of treatment administration, level of gregariousness of the animal used, phylogenetic relatedness between the animal used and human patients, and immunocompetency levels of the patients exposed to zootherapeutic practices.

## 2 Methods and design

### 2.1 Systematic review

Following the updated PRISMA guidelines [23] we conducted a systematic review of literature published until 30th of July 2023, using web-scraping algorithms targeted at peer-reviewed (PubMed) and peer-reviewed or grey literature (Google Scholar) databases, followed by a manual search of reference lists. We also obtained publications and master/doctoral theses from the main organisation affiliated with this study (Royal Belgian Institute of Natural Sciences, Brussels, Belgium). Terms encompassing zoo*, animal*, health*, practice*, tradition* were used in combination with Boolean operators for all 54 African countries, following the search string ‘(Country Name) AND (zoo* OR animal*) AND (health* OR practice* OR tradition*)’.

Following quality analysis using the MMAT framework [24] (Additional file 1), studies were included if they were published in peer-reviewed journals or as scientific theses in French or English after 1990, and if they explicitly stated the name of the animal and the ailment treated, the animal tissue type used, and/or the treatment method. Three independent reviewers retrieved (AL, LF, GTS), screened (LF, GTS) and assessed (LF, GTS) the studies. In addition to the extraction of metadata, a data extraction template was created with five main sections, focusing on: the animal (genus, species), the tissue type, the method of treatment administration, and the human patient demographic category.

We then investigated the geographic and temporal variations in the recorded zootherapeutic practices by mapping the number of studies and total study size in each country, and by constructing saturation curves of the cumulative number of distinct practices recorded with each new study. All code is available via the linked github repository (https://github.com/dimitri009/Zootherapy).

### 2.2 Selection of the risk factors and risk analysis

We assessed the risk of zoonotic pathogen spillover for each distinct zootherapeutic practice using a scoring system that reflects the likelihood of the successful establishment of any pathogen in the human patient after performing this zootherapeutic practice. Using peer-reviewed evidence, we first identified risk factors suggested to contribute to zoonotic spillover risk (Additional file 2), which is characterised by the ability of an animal-sourced pathogen to infect and cause disease in humans. Specifically, we followed a conceptual approach where factors of importance in the likelihood of spillover are divided into ranked classes, where each rank obtains a value (1–5), five representing the highest risk [3].

Commonly cited risk factors included the phylogenetic relatedness between the animal species and humans ([25] but see [26] for nuances), the level of gregariousness of the animal species [25] and the immunocompetence of the human recipient at the time of the treatment [18,27]. We also chose to include the animal tissue type that the patient would be in contact with (e.g., bones, fur, blood), and the method of treatment administration (e.g., ingestion after cooking, inhalation, topical application on a wound), as pathogens are present at higher concentrations in certain tissues, and are more likely to reach human cells through certain exposure routes [18]. We then scored the components making up each of these risk factors, from five (highest risk of zoonotic pathogen spillover) to one (lowest risk), as shown in Table 1 and Additional file 2. The total risk score was created by summing the scores across factors for each practice.

**Table 1:** Categorical components of the risk factors.

Factor/Score	Tissue type	Treatment type	Phylogenetic relatedness	Gregariousness	Immunocompetence (Human recipient)
Highest risk (5)	Blood, internal organs, whole animal (including foetus)	Injected, topical (raw) on wound	Primates	Social	Physically sick child or infant
High risk (4)	Faeces, urine, secretions (vaginal, seminal, saliva), milk, flesh, fat, skin, eggs	Spraying/pouring^1^ inhalation (raw), ingestion (raw), topical (raw) on mucosa, topical (altered/cooked) on wound	Other mammals	NA	Seemingly physically healthy child or infant (psychological or spiritual issues, or preventive medicine)
Medium risk (3)	Bones	Ingestion (altered)^2^, topical, topical (altered/cooked)^2^ on mucosa	Birds	Restricted family unit or seasonally social	Pregnant or lactating woman
Low risk (2)	Hair/fur, feathers	Inhalation (altered)^2^	Reptiles, amphibians, fish	NA	Physically sick adult
Lowest risk (1)	Honey, butter, scales, nails, horns, sting, venom, teeth	Ingestion (cooked)	Invertebrates	Solitary	Seemingly physically healthy adult (psychological or spiritual issues, or preventive medicine)

1 ‘Spraying/pouring’ refers to animal tissues being sprayed or poured around the patient (e.g.*,* on the walls or floor near the patient).

2 ‘Altered’ can be dried, sun-dried, crushed to powder, smoked, or a combination of these.

To examine the risk score across geographic regions, we used the total risk score, where all five factors are included (maximal possible total score of 25). To examine the risk score across demographic groups, we used a sub-score that excludes immunocompetency, as immunocompetency correlates with the demographic categories of interest for this study (maximal possible sub-score of 20). To investigate whether the risk score varied across geographic regions or demographic categories, we conducted pairwise comparisons using Kruskal–Wallis testing, followed by a Bonferroni-correction.

## 3 Results

### 3.1 Spatial and temporal trends in primary research

**Fig. 1:**
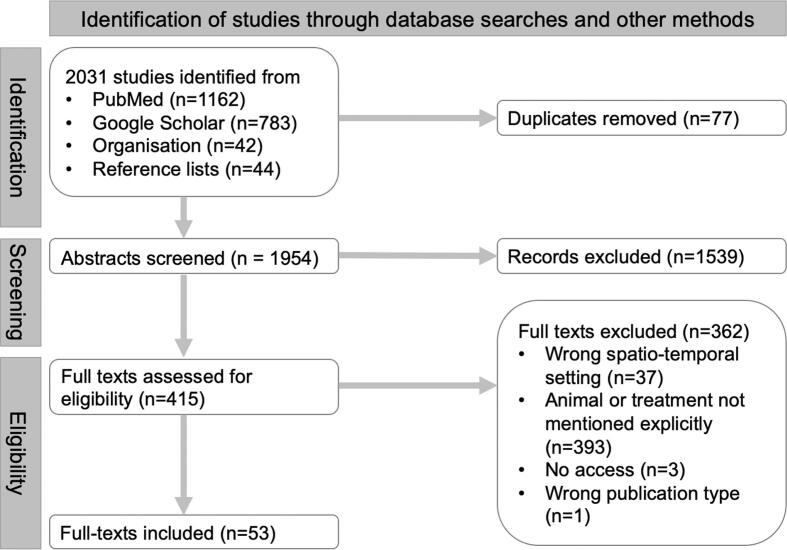
PRISMA flow chart highlighting search outcomes.

**Fig. 2:**
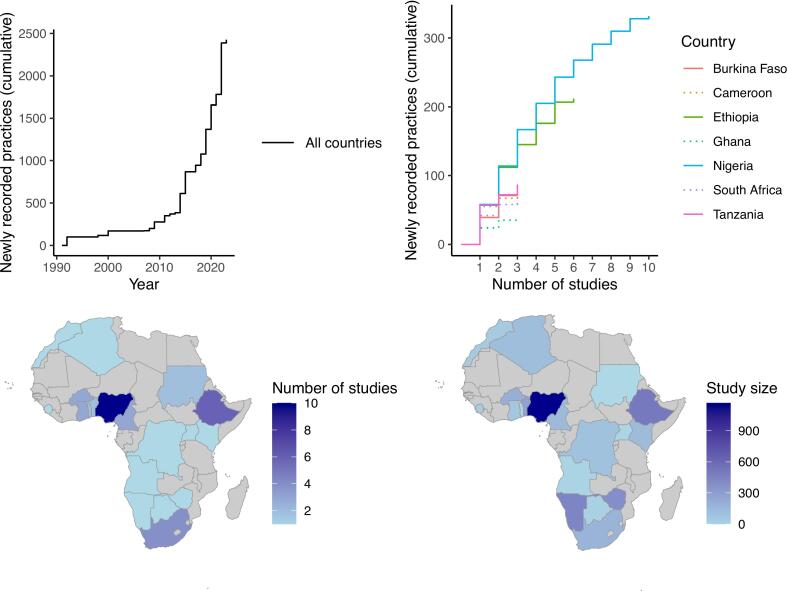
Spatial and temporal trends in studies on African zootherapeutic practices. A: number of new practices recorded since 1990. B: saturation curve representing the number of new practices recorded with each new study (the graph shows a subset of the seven countries having the greatest number of studies). C: number of studies published for each country. D: overall study size for each country (all studies included).

Of the 2031 records retrieved, 415 full-texts were assessed for eligibility, and 53 studies met all the inclusion criteria (Fig. 1 and Additional file 1). Half of the 53 included studies were published in or after 2020, leading to a stark temporal increase in the number of newly recorded practices, and 37 studies were led by African scholars (Fig. 2A, Additional file 1).

With ten studies published in the last thirty years, Nigeria had both the highest number of publications and the highest overall study size (Fig. 2B, C and D). The saturation curve indicates that the number of new practices recorded in each new study started to plateau in Nigeria, with no plateau in other countries yet (Fig. 2B).

### 3.2 Characteristics and spatial distribution of spillover risk

**Fig. 3:**
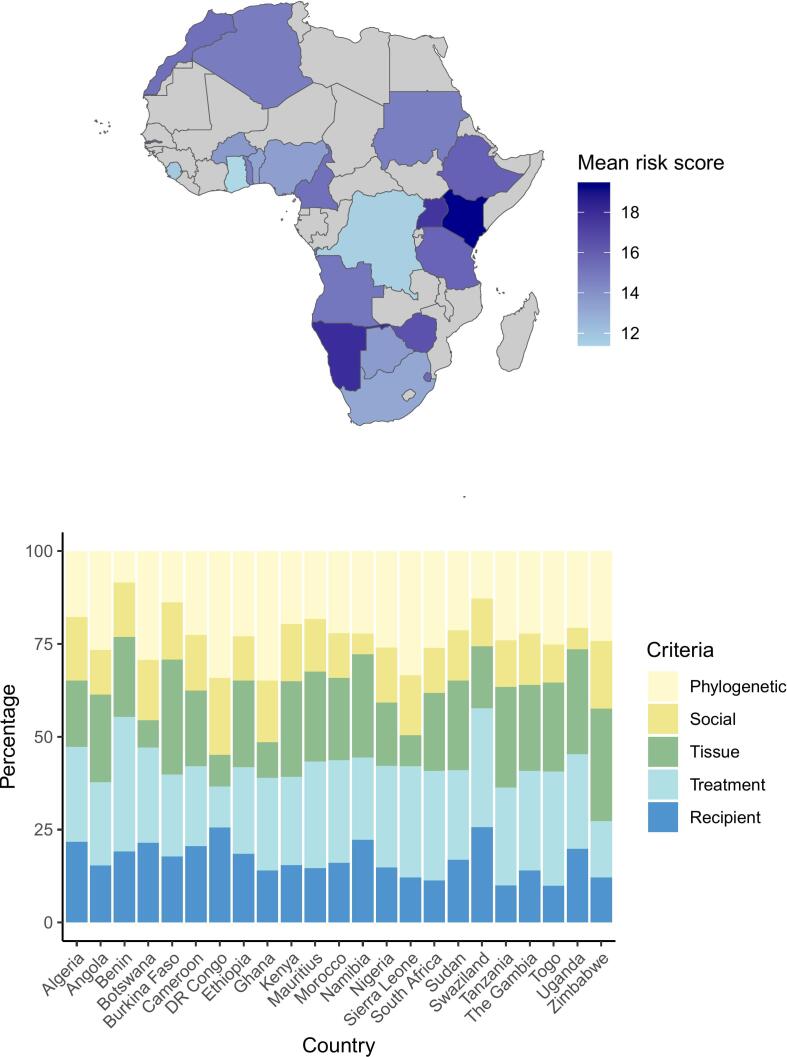
Spatial distribution of risk. A: risk map. B: stacked bar plot showing the drivers of risk (%) for each country. DR Congo: Democratic Republic of Congo.

Overall, the mean total risk of zoonotic pathogen spillover was moderate (14.00 ± 2.80 out of a maximum possible total score of 25, *n* = 2425). The treatment type was a major contributor to risk, accounting for 20 % or more of the risk score in 21 out of 23 countries (Fig. 3). Phylogenetic relatedness between humans and animals used as the source of the treatment, was also an important contributor to risk in most countries, accounting for 20 % or more of the risk score in 18 out of 23 countries. Exceptions include Benin, where phylogenetic relatedness minimally contributed to risk; and D.R. Congo and Zimbabwe, where the method of treatment minimally contributed to risk (Fig. 3B).

**Fig. 4:**
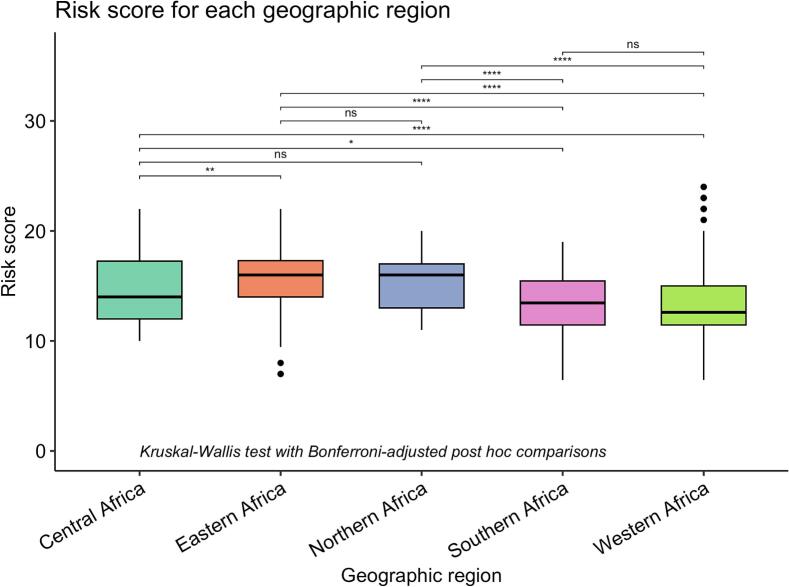
Differences in risk scores attributed to practices in each geographic region. ns: non-significant, * : p-val <0.05; ** : p-val < 0.01; *** : p-val <0.001; **** : p-val >0.0001.

We found regional differences in the risk of zoonotic pathogen spillover. Practices occurring in eastern Africa (mean = 15.6, ± 2.59) and northern Africa (mean = 15.2, ± 2.39) had the highest average risk score. There was no significant difference in risk score between practices occurring in northern or eastern Africa (H = 1.26, *p* = 0.21, p-adj = 1.00), both regions having the highest average risk scores. Similarly, there was no significant difference between risk for practices occurring in western or southern Africa (H = 1.27, *p* = 0.20, adj-*p* = 1.00), both regions having the lowest risk scores (Table S1). However, the analysis revealed that the risk scores attributed to practices were otherwise significantly impacted by location, with scores linked to practices occurring in eastern Africa being significantly higher than those of practices occurring in central Africa (H = 3.35, *p* = 0.0008, p-adj = 0.008), western Africa (H = 17.2, *p* = 2.5e-66, p-adj = 2.5e-65), and southern Africa (H = 8.47, *p* = 2.55e-17, p-adj = 2.55e-16), indicating a significant distinction in risk based on the patient's geographic location (Fig. 4, Table S1).

### 3.3 Demographic distribution of spillover risk

**Fig. 5:**
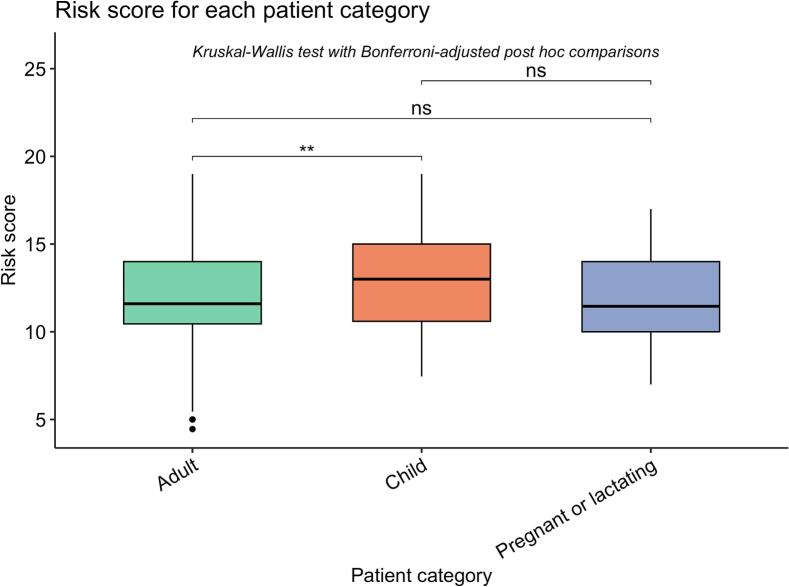
Differences in risk scores attributed to practices targetted at each patient category. ns: non-significant; ** : p-val <0.01.

The analysis revealed that the risk score attributed to practices targeted at children was significantly higher than those targeted at adults (H = 3.25, *p* = 0.001, p-adj = 0.003), indicating a significant distinction in risk based on the patient's age category (Table S2, Fig. 5).

Contrarily, there was no significant difference between adults and pregnant or lactating individuals (*p* = 0.948, p-adj = 1.00), nor between children and pregnant or lactating individuals following Bonferroni's correction (*p* = 0.036, p-adj = 0.108), despite the mean score for the pregnant/lactating patients being numerically lower (Table S2, Fig. 5).

## 4 Discussion

### 4.1 Publication landscape

Our analysis identified trends in the publication landscape regarding primary research on zootherapeutic practices. We found that this topic is gaining traction, with half of the included studies being published in the last five years. Likely drivers of this surge in interest encompass recently increased research efforts in the domain of zoonoses, to better understand spillover mechanisms (e.g. [17]); and the increased attention given to Traditional, Complementary and Integrative Medicine by the World Health Organisation and other agencies to reach the Sustainable Development Goal 3: Good Health and Wellbeing [28].

This growing interest in traditional ethnomedical practices offers an opportunity to strengthen the currently limited record of zootherapeutic traditions across Africa, laying the foundation to quantify risks and benefits at the community level, and, subsequently, for conducting meta-analyses at the national and regional level. Continued research will be essential to set a scientific standard that will allow the integration of the most effective traditional practices into contemporary medicine, in line with the P5 movement, which promotes preventive, personalized, participatory, precise, and predictive medical practices [29].

Additionally, we found that over two thirds of studies were led by scholars affiliated to research groups of the countries where the study took place, highlighting a strong local expertise and research interest (Additional file 1). Furthermore, most studies were conducted through collaborations between multiple institutes, often from several countries, highlighting that researchers and administrations are aware that local traditional practices may have extensive repercussions, either positive or negative, beyond the local context [30].

Except for Nigeria, no country has reached a plateau in reporting new practices, highlighting the need for further research across Africa (Fig. 1). In Nigeria, recent studies rarely reveal previously undocumented practices, whereas in other countries, each new study is likely to reveal several. However, even in Nigeria, new practices may still emerge, warranting continued monitoring. Overall, priority should be given to regions lacking recent peer-reviewed data, including the Sahel region (e.g., Mauritania, Mali, Niger, Chad, Senegal), parts of Central Africa (e.g., Gabon, Central African Republic), and parts of Eastern and Southern Africa (e.g., Malawi, Mozambique, Madagascar).

### 4.2 Geographic and demographic distribution of risk

Regarding the geographic distribution of risk, the inferred risk score was significantly lower in western, central, and southern Africa, compared to eastern and northern Africa. This observation may be linked to the frequent use of animal products from domesticated animals in eastern Africa, which provide easy access to tissues of greater infectious potential, such as milk, blood, semen, or saliva. Ethnicities for which the raw blood and milk of ungulates is a key component of physical and spiritual strength, such as the Maasai or Rendile, may be exposed (and expose visitors and travellers participating in such rituals, see [31]) to zoonotic pathogens causing several severe diseases, including Q-fever, anthrax, bovine tuberculosis and brucellosis [8]. This is especially the case where people believe in the purity of their cattle's blood, meat and milk, leading to the preference for raw products [8] and consumption even if the animal showed signs of disease [32]. Veterinary services, in cooperation with traditional healers and community leaders in eastern Africa therefore have an important role to play [8]. Similar comments can be made for north-African countries, where the importance of herding and cultural specificities stemming from a nomadic heritage can also facilitate the reliance on animal products by certain ethnicities, such as the Touaregs, to combat ailments in historically hot and dry environments [33]. Water scarcity may also discourage the boiling of products in these regions, favouring the use of raw, rather than heat-treated, products.

Importantly, while having a lower overall risk score, several high-risk practices were recorded in western and central Africa, especially regarding the phylogenetic relatedness between the animal and the human patient (e.g., reliance on primate products, such as gorillas or mandrills). Further, in western and central Africa, the widespread belief that wild animals are healthier than their domesticated counterparts [34] may trigger the spillover of wildlife pathogens unknown to western science, and for which western treatments, such as antimicrobial drugs or vaccines, are not available. The knowledge that wild animals may propagate pathogens causing severe diseases, such as haemorrhagic fevers, is not a sufficient deterrent to overcome the cultural (and monetary) importance of wild animals [35] even for the west-African diaspora [36]. As such, despite their lower overall risk score, these regions also warrant a detailed examination and monitoring of their practices.

We then uncovered demographic trends in pathogen spillover risk associated with zootherapeutic practices. Our results indicate that vulnerable population members, especially children, are at an overall greater risk of zoonotic pathogen spillover through zootherapeutic practices than adults, mostly because of treatment methods of greater infectious potential. Examples include the injection of fluids containing *Mandrillus* sp. bones or *Gorilla* sp. bones to prevent “weakness” in children. Practices concerning newborn and paediatric health can be even more culturally sensitive than practices targeting adult patients, especially in cultures where children are thought to impersonate reborn ancestors. Therefore, belief systems linked to ethnopaediatrics deserve special attention from researchers [37]. This is especially important, given variations in the immunocompetency of these demographic groups [27]. As such, traditional paediatric and maternal care practices should be carefully monitored, and direct contact between infants and raw animal products should generally be discouraged, while ensuring meaningful alternatives can be elaborated.

Further, given the high HIV burden in numerous African states [38] it is likely that many zootherapeutic treatments are administered to HIV-positive people without always explicitly being sold as such, either to cure HIV itself, or symptoms of HIV, or because of unrelated symptoms. Our dataset comprises four practices explicitly aimed at curing HIV, for instance through the ingestion of dried hippopotamus blood in Tanzania [39]. The administration of zootherapeutics to immunodepressed patients should generally be discouraged, as the risks of secondary infections are high compared to any potential benefits.

### 4.3 Other risk factors

Lastly, we want to point out that the risks linked to zootherapeutic practices can be made worse by at least two other factors: antimicrobial resistance and hazardous preservatives. Antimicrobial-resistant bacteria have already been found in animal *materia medica*, for instance Bacillus spp. (100 % AMR) and *Staphylococcus* spp. (12.5 % AMR) in cow urine used for zootherapy in Nigeria [40]. We also suspect that pastoralist communities may be more exposed to resistant bacteria when herders give antimicrobial drugs to cattle without proper monitoring—especially given the belief that the meat should not be wasted even if the animal was slaughtered or died shortly after receiving the drugs [32,41,42].

Hazardous preservatives can add another layer of risk [43]. While adding salt or beach sand might seem harmless (if sterilized), the use of kerosene, insecticides, or formalin is far more concerning. Kerosene and insecticides are particularly common—used by 98 % and 71 % of respondents, respectively—for getting rid of microorganisms and keeping products for longer. This means that the danger from pathogens could be compounded by the toxic effects of the added chemicals, affecting both the end-user and the seller [43]. It is thus imperative to assess the risks and benefits of TCIM in a more comprehensive manner for TCIM to genuinely contribute to better health globally.

### 4.4 Caveats

This study has the following limitations: First, we included articles where taxonomic identification of the animal was sometimes limited to the genus, whereas species-level or even subspecies-level identifications would provide more information. Where possible, we encourage interdisciplinary collaborations to ensure precise taxonomic identifications. As several African taxa are currently undergoing taxonomic revisions (e.g.*, Sciuridae* spp. or *Soricidae* spp.), future meta-analyses should account for such changes. Second, most studies did not detail ages and genders at which the practices are targeted. As such, we created overarching categories such as ‘children’ or ‘pregnant or breastfeeding woman’ without accounting for differences between the immunological capacities of newborns, infants, and children; or at different stages of pregnancy and *post-partum*. We thus invite researchers to collect and publish more precise and specific data about the patients. Last, we conducted analyses at national and regional levels. However, some practices may only be applicable to very localized ethnicities, hence not being representative of a country. Contrarily, other practices may be shared by multiple ethnicities, or ethnicities that may inhabit several countries, including countries not mentioned in the study where the practice is described. As such, results should be interpreted where culturally relevant.

## 5 Recommendations

Our general recommendation is to encourage a pan-African reflection on the risks and benefits of traditional medicine, for which the creation of a pan-African database reporting zootherapeutic practices would be helpful. This could include i) the animal species used, ii) the animal product type used (e.g., faeces, blood, bones, hairs, skin), iii) whether and how the product is treated before being administered (e.g., boiled, sun-dried, raw), iv) how the product is administered (e.g.*,* ingestion, topical application, inhalation), v) who the product is aimed at (e.g., age, sex, medical background), and vi) against which type of ailment or for which benefit (e.g.*,* injury, disease, mental health, preventive medicine). Additional notes on location, seasonal use or taboo would be helpful to best determine spillover risk, as the circulation of several pathogens (e.g., RNA-viruses) depends on the breeding season of the animal host, which in turn depends on the local geography. Similarly, notes on chemical preservatives, additives, or other products added to the preparation could be useful to evaluate possible interactions between key compounds.

Such a database could be fuelled both by local communities and researchers, with support from official administrations. To minimise reluctance from traditional practitioners to share information, the recipe itself (i.e.*,* the quantities of product used, or any ritual performed during the preparation of the product) should remain undisclosed. Notably, several other cultures have published their traditional practices in referenced textbooks, which can be used to streamline comparison across ethnicities (see e.g., [44]). In addition to offering a quality resource to monitor risk, such a database would thus also improve the documentation and safeguarding of these traditions in a rapidly changing world.

Further, we have three sets of specific recommendations, aimed at researchers, health practitioners, and local communities, respectively. First, we encourage interdisciplinary collaboration between social scientists, public health researchers, biologists, and veterinarians in the collection and publication of data regarding zootherapeutic practices, to ensure the categories described above are documented as accurately as possible. For numerous countries (e.g.*,* Sengal, Niger, Malawi, Mozambique, Gabon, Liberia, Somalia), we have found no recent publication on zootherapeutic practices. While this could indicate that only plants or minerals are traditionally used by local communities, we believe that further research should be conducted to ensure that the lack of publication is not a false negative.

Second, where possible, official health centres should enquire whether, and which, traditional practices have been trialled before reaching out to governmental or NGO-based healthcare, and whether patients have experienced different symptoms following such a traditional practice. Collaboration, in person or through telephone, between official health centres and local practitioners should be encouraged to allow local practitioners to share records of the conditions they have treated, and of the outcome of the treatment they have administered. These records should then be analysed, to quantify the possible benefits and adverse effects of these practices (e.g., [45]). Additionally, local practitioners could be encouraged to try floral or mineral alternatives to zootherapeutics, where the zootherapeutic practice entails a high risk of spillover.

Last, we recommend engaging customers themselves, both by informing them of possible risks, and by being informed by them of their preferred treatments. This could lead to a switch in demand, favouring lower-risk practices, where animals are phylogenetically distant from humans (e.g., invertebrates, reptiles or fish rather than mammals), their products contain few bodily fluids (e.g., claws, scales, or feathers rather than blood or faeces), undergo heat treatment (e.g., boiling, or sun-drying rather than raw consumption), and treatment administration limits possibly harmful contact (e.g.*,* topical application on a healthy skin rather than ingestion, inhalation, injection or application on a wound). People whose immune system might be compromised through disease, age, or pregnancy, should be made especially aware of spillover risks.

As such, local communities, their traditional practitioners, and official health centres should discuss whether, and how, to best adapt the riskiest traditions, so that culturally appropriate alternatives exist to satisfy the needs of local communities as safely as possible.

## 6 Conclusion

Traditional medical practices are a key component of spiritual and physical health for a majority of Africans. Nonetheless, the use of animal products as *materia medica* can entail public health risks by facilitating the spillover of zoonotic pathogens. This article aimed at reviewing zootherapeutic practices and analysing the public health risk they entail for the African continent.

We first conducted a systematic review of literature, before creating a categorical score reflecting the risk of zoonotic pathogen spillover for each recorded practice. We found that half of the studies included in our analysis were published between 2020 and 2023, indicating growing interest in the topic since the COVID-19 pandemic. Mean risk scores were significantly higher for practices in eastern and northern Africa compared to those in central, western, and southern regions—likely linked to herding traditions in the north and east, which increase access to high-risk animal products such as raw blood, milk, and faeces. In these areas, (ethno-)veterinarians have a crucial role in working with local herders to co-develop safer alternatives where appropriate.

Although western Africa had a lower average risk score, certain regional practices—particularly those involving phylogenetically close species such as primates—carry high risk and warrant closer investigation and monitoring. We also found that children face a higher overall risk of pathogen spillover than adults. This is a particularly sensitive issue, as some communities may resist modifying or discontinuing ethnomedical practices for children due to spiritual beliefs or the desire to preserve ancestral connections.

We thus encourage local communities, traditional health practitioners, researchers, and government-led health administrations to collaborate on (i) the elaboration of a pan-African collection of traditional practices, to better identify and maintain the cultural heritage linked to such practices; (ii) the regular monitoring of risks and benefits linked to such practices, including any comorbidities linked to hazardous preservatives, and with a special focus on the spillover of anti-microbial resistant pathogens; and (iii) the elaboration of culturally meaningful alternatives to the practices that entail higher risks than benefits, to satisfy local demands without endangering the physical health of patients and their communities.

## CRediT authorship contribution statement

**Léa Fourchault:** Writing – review & editing, Writing – original draft, Visualization, Resources, Project administration, Methodology, Investigation, Funding acquisition, Formal analysis, Data curation, Conceptualization. **Abdallah Lamane:** Writing – review & editing, Visualization, Software, Investigation, Formal analysis. **Nguinwa Mbakop Dimitri Romaric:** Writing – review & editing, Visualization, Formal analysis, Data curation. **Ganiyat Temidayo Saliu:** Writing – review & editing, Investigation, Data curation. **Sophie Gryseels:** Writing – review & editing, Validation, Supervision, Resources, Project administration, Methodology, Investigation, Funding acquisition. **Erik Verheyen:** Writing – review & editing, Supervision, Project administration, Methodology, Funding acquisition. **Farid Dahdouh-Guebas:** Writing – review & editing, Supervision, Funding acquisition. **Katharina Kreppel:** Writing – review & editing, Validation, Supervision, Resources, Project administration, Methodology, Investigation, Conceptualization.

## Declaration of competing interest

The authors declare that they have no known competing financial interests or personal relationships that could have appeared to influence the work reported in this paper.

## Data Availability

All data is being deposited, along with the code, on Github at https://github.com/dimitri009/Zootherapy. Both first and last authors can be contacted for further details.

## Appendix A Supplementary data

Supplementary material 1 Image 1

Supplementary material 2 Image 2

Supplementary material 3 Image 3
