# Supplementary material for: Public health risks of traditional zootherapeutic practices in Africa
Source: One Health. 2025 Aug 20;21:101178. doi: 10.1016/j.onehlt.2025.101178 (PMC12765085; doi:10.1016/j.onehlt.2025.101178)
Supplement: Supplementary file 1 — Supplementary material 1 [file mmc1.docx]

**Table S1. Comparison of risk scores across geographic regions (Kruskal-Wallis test followed by post-hoc pairwise analysis with Bonferroni correction).**

| **Group 1** | **Group 2** | **H-statistic** | **p-value** | **Adjusted p-value** | **Significance** |  |
| --- | --- | --- | --- | --- | --- | --- |
| Central Africa | Eastern Africa | 3,352783 | 0,0008 | 0,008 | ** |  |
| Central Africa | Northern Africa | 1,685539 | 0,091885 | 0,918846 | ns |  |
| Central Africa | Southern Africa | -3,20926 | 0,001331 | 0,013308 | * |  |
| Central Africa | Western Africa | -5,0287 | 4,94E-07 | 4,94E-06 | **** |  |
| Eastern Africa | Northern Africa | -1,26176 | 0,207034 | 1 | ns |  |
| Eastern Africa | Southern Africa | -8,46568 | 2,55E-17 | 2,55E-16 | **** |  |
| Eastern Africa | Western Africa | -17,2036 | 2,5E-66 | 2,5E-65 | **** |  |
| Northern Africa | Southern Africa | -5,15653 | 2,52E-07 | 2,52E-06 | **** |  |
| Northern Africa | Western Africa | -7,58479 | 3,33E-14 | 3,33E-13 | **** |  |
| Southern Africa | Western Africa | -1,26953 | 0,204251 | 1 | ns |  |

ns: non-significant; * : p-val < 0.05; ** : p-val <0.01; *** : p-val <0.001; **** : p-val <0.0001.

**Table S2. Comparison of risk scores across patient categories (Kruskal-Wallis test followed by post-hoc pairwise analysis with Bonferroni correction).**

| **Group 1** | **Group 2** | **H-statistic** | **p-value** | **Adjusted p-value** | **Significance** |
| --- | --- | --- | --- | --- | --- |
| Adult | Child | 3,249555 | 0,001156 | 0,003468 | ** |
| Adult | Pregnant or lactating | 0,065725 | 0,947596 | 1 | ns |
| Child | Pregnant or lactating | -2,09793 | 0,035912 | 0,107735 | ns |

ns: non-significant; ** : p-val <0.01.
