# Supplementary material for: Public health risks of traditional zootherapeutic practices in Africa
Source: One Health. 2025 Aug 20;21:101178. doi: 10.1016/j.onehlt.2025.101178 (PMC12765085; doi:10.1016/j.onehlt.2025.101178)
Supplement: Supplementary file 2 — Supplementary material 2 [file mmc2.docx]

**Public health risks of traditional zootherapeutic practices in Africa**

Léa Fourchault^a,b^, Abdallah Lamane^a,c^, Nguinwa Mbakop Dimitri Romaric^a,d^, Ganiyat Temidayo Saliu^a^, Sophie Gryseels^a,e^, Erik Verheyen^a,e^, Farid Dahdouh-Guebas^b,f,g^ Katharina Kreppel^h^

^a^ *Operational Directorate Taxonomy and Phylogeny, Royal Belgian Institute of Natural Sciences, Brussels 1000, 29 Vautierstraat, Belgium*

^b^ *Systems Ecology and Resource Management, Université libre de Bruxelles, 50 Avenue Franklin D. Roosevelt, CPI 264/1 B-1050 Brussels, Belgium*

^c^ *CentraleSupélec, Université Paris-Saclay, Gif-sur-Yvette 91192, 3 rue Joliot-Curie, Fance*

^d^ *Department of Computer Engineering, Università degli Studi di Firenze, Firenze 50121, 4 Piazza San Marco, Italy*

^e^ *Evolutionary Ecology Group (EVECO), University of Antwerp, Antwerp 2000, 13 Prinsstraat, Belgium*

^f^ *Ecology, Evolution & Genetics Research Group (bDIV), Department of Biology, Vrije Universiteit Brussel - VUB, Pleinlaan 2, B-1050, Brussels, Belgium*

^g^ *Mangrove Specialist Group (MSG), Species Survival Commission (SSC), International Union for the Conservation of Nature (IUCN), c/o Zoological Society of London,*

*London, United Kingdom*

^h^ *Emerging Infectious Diseases Unit,* *Antwerp Institute for Tropical Medicine, Antwerp 2000, 155 Nationalestraat, Belgium* ([kkreppel@itg.be](mailto:kkreppel@itg.be))

*Corresponding author’s full contact details: [lfourchault@naturalsciences.be](mailto:lfourchault@naturalsciences.be); Royal Belgian Institute of Natural Sciences, 27 Vautierstraat, Brussels 1000, Belgium; +33 609449778

**Choice of risk categories and rankings therein**

The risk of spillover resulting from each zootherapeutic practice was scored using a point system (1-5, where higher points are given to riskier practices), where points were summed across five categories: (i) phylogenetic relatedness between the human patient and the animal used in the practice, where closer relatedness leads to higher risk; (ii) the level of gregariousness of the animal used in the practice, where more social animals carry higher risk; (iii) the immunocompetence of the human patient; (iv) the type of treatment, such as ingestion or topical application and (v) the type of animal tissue used, such as blood, meat or feaces.

1. **Phylogenetic relatedness**

The relationship between phylogenetic relatedness and pathogen spillover remains complex (e.g., Becker *et al*., 2019), but evidence to date implies that evolutionary constraints limit cross-species transmission and host-switching (e.g., Brooks, 1988; Poulin, 2010; McKee *et al.,* 2016; Clark & Clegg, 2017; Engelstädter & Fortuna, 2019; Shaw *et al*., 2020), even in pathogens exhibiting high mutational rates, such as RNA viruses (Streicker *et al*., 2010). When considering viruses, bacteria, helminths, protozoans, and fungi, about 11% of rodent species carry zoonotic pathogens, including 85 unique zoonoses; about 10% of bat species carry zoonotic pathogens, including 27 unique zoonoses; about 4% of shrew species carry zoonotic pathogens, with 19 unique zoonoses (*nb*, there are 1-2 orders of magnitude fewer studies on shrews compared to bats and rodents); about 50% of carnivore species carry zoonotic pathogens, including 83 unique zoonoses; about 32% of ungulate species carry zoonotic pathogens, including 68 unique zoonoses; and about 21% of primate species carry zoonotic pathogens, including 63 unique zoonoses (Han *et al*., 2016; corroborated by Moloney *et al*., 2023). Therefore, while primates, carnivores and ungulates are less speciose than rodents, shrews and bats, they harbour a greater proportion of zoonotic pathogen host species.

Among primates, carnivores, and ungulates, primates are most closely related to humans, and were thus given the highest ranking, following the hypothesis that phylogenetic relatedness influences the likelihood of host-switching due to a smaller ‘jump’ distance between hosts (e.g., Brooks, 1988; Poulin, 2010; McKee *et al*., 2016; Clark & Clegg, 2017), with up to a third of variation in pathogen communities being explained by phylogenetic distance when considering primates (e.g., Petersen and Davies, 2009). Other mammals were given the second-highest ranking, as current literature does not allow to discriminate between the likelihood of spillover from the superorders Euarchontoglires (primates, rodents, lagomorphs) versus Laurasiathera (e.g., bats, carnivores), wherefore we chose to remain at the level of the order, and to discriminate only between primates and all other mammalian orders.

**References**

Becker, D. J., Washburne, A. D., Faust, C. L., Mordecai, E. A., & Plowright, R. K. (2019). The problem of scale in the prediction and management of pathogen spillover. *Philosophical Transactions of the Royal Society B*, *374*(1782), 20190224.

Brooks, D. R. (1988). Macroevolutionary comparisons of host and parasite phylogenies. *Annual Review of Ecology and Systematics*, 235-259.

Clark, N. J., & Clegg, S. M. (2017). Integrating phylogenetic and ecological distances reveals new insights into parasite host specificity. *Molecular Ecology*, *26*(11), 3074-3086.

Engelstädter, J., & Fortuna, N. Z. (2019). The dynamics of preferential host switching: host phylogeny as a key predictor of parasite distribution. *Evolution*, *73*(7), 1330-1340.

Han, B. A., Kramer, A. M., & Drake, J. M. (2016). Global patterns of zoonotic disease in mammals. *Trends in parasitology*, *32*(7), 565-577.

McKee, C. D., Hayman, D. T., Kosoy, M. Y., & Webb, C. T. (2016). Phylogenetic and geographic patterns of bartonella host shifts among bat species. *Infection, Genetics and Evolution*, *44*, 382-394.

Moloney, G. K., Gaubert, P., Gryseels, S., Verheyen, E., & Chaber, A. L. (2023). Investigating infectious organisms of public health concern associated with wild meat. *Transboundary and Emerging Diseases*, *2023*(1), 5901974.

Pedersen, A.B., Davies, T.J. (2009). Cross-Species Pathogen Transmission and Disease Emergence in Primates. *EcoHealth* *6*, 496–508.

Poulin, R. (2010). Decay of similarity with host phylogenetic distance in parasite faunas. *Parasitology*, *137*(4), 733-741.

Shaw, L. P., Wang, A. D., Dylus, D., Meier, M., Pogacnik, G., Dessimoz, C., & Balloux, F. (2020). The phylogenetic range of bacterial and viral pathogens of vertebrates. *Molecular ecology*, *29*(17), 3361-3379.

Streicker, D. G., Turmelle, A. S., Vonhof, M. J., Kuzmin, I. V., McCracken, G. F., & Rupprecht, C. E. (2010). Host phylogeny constrains cross-species emergence and establishment of rabies virus in bats. *Science*, *329*(5992), 676-679.

**2a. Level of gregariousness**

Increased pathogen prevalence is generally regarded as a cost of gregariousness, where group formation and greater group size are drivers of parasitic infection (Altizer *et al*., 2003; Rifkin *et al.,* 2012; Patterson and Ruckstuhl, 2013; Kappeler *et al*., 2015; VanderWaal *et al*., 2016), even if sociality can also decrease the spread of pathogens, for instance through behavioural adaptation, herd immunity, or subpopulation structures (Nunn *et al*., 2015; Kappeler *et al*., 2015; Ezenwa *et al*., 2016). Here, we categorised species as either solitary, e.g., *Python sebae* (lowest risk, one point), occasionally social or living in restricted family units, e.g., *Tragelaphus scriptus* (medium risk, three points), or as social, e.g., *Bos taurus*, (highest risk, five points), where multiple family units share space and resources throughout the year. To determine the level of gregariousness for each species, we consulted scientific literature and summaries from the Animal Diversity Web. Domestic animals were ranked as social (highest risk), except for *Felis domesticus*, which was ranked as occasionally social (medium risk). The mean gregariousness score obtained across our dataset (*i.e*., 2.6, n = 143) was attributed to animals of whom the level of gregariousness could not be determined effectively (e.g., *Eudrilus eugeniae*).

**References**

Altizer, S., Nunn, C. L., Thrall, P. H., Gittleman, J. L., Antonovics, J., Cunningham, A. A., ... & Pulliam, J. R. (2003). Social organization and parasite risk in mammals: integrating theory and empirical studies. *Annual Review of Ecology, Evolution, and Systematics*, *34*(1), 517-547.

Ezenwa, V. O., Ghai, R. R., McKay, A. F., & Williams, A. E. (2016). Group living and pathogen infection revisited. *Current Opinion in Behavioral Sciences*, *12*, 66-72.

Kappeler, P. M., Cremer, S., & Nunn, C. L. (2015). Sociality and health: impacts of sociality on disease susceptibility and transmission in animal and human societies. *Philosophical Transactions of the Royal Society B: Biological Sciences*, *370*(1669), 20140116.

Nunn, C. L., Jordán, F., McCabe, C. M., Verdolin, J. L., & Fewell, J. H. (2015). Infectious disease and group size: more than just a numbers game. *Philosophical Transactions of the Royal Society B: Biological Sciences*, *370*(1669), 20140111.

Patterson, J. E., & Ruckstuhl, K. E. (2013). Parasite infection and host group size: a meta-analytical review. *Parasitology*, *140*(7), 803-813.

Rifkin, J. L., Nunn, C. L., & Garamszegi, L. Z. (2012). Do animals living in larger groups experience greater parasitism? A meta-analysis. *The American Naturalist*, *180*(1), 70-82.

VanderWaal, K. L., Obanda, V., Omondi, G. P., McCowan, B., Wang, H., Fushing, H., & Isbell, L. A. (2016). The “strength of weak ties” and helminth parasitism in giraffe social networks. *Behavioral Ecology*, *27*(4), 1190-1197.

**2b. Level of gregariousness versus wild/domestic**

While previous studies have discriminated between domestic and wild species regarding the likelihood of pathogen spillover (e.g., Olival *et al*., 2017), extensive farming dominates in Africa, where domestic and wild animals are rarely physically separated, and can therefore exchange pathogens through both direct and indirect contact (e.g., Mitchell, 2018). Numerous pathogens have been recorded in both wild and domestic animals throughout the African continent (Ohiolei *et al*., 2020; Calkins and Scasta, 2020), and the distinction between wild and domestic can be blurred. Indeed, some ‘wild’ animals may be kept and bred as domestic animals commonly (e.g., cane rats or porcupines in Ivory Coast; primates kept and sold as pets around the world, Muehlenbein, 2017) or kept in close contact with domestic animals (e.g., game animals foraging alongside cattle in Namibia; Kurpiers *et al*., 2016); while domestic animals may be left to forage outside of the domestic sphere for extended time periods (e.g., cattle in Kenya). As it was not always possible to identify whether the animals cited in our studies were kept within the domestic sphere prior to their use, we chose not to include this distinction in our risk analysis.

**References**

Calkins, C. M., & Scasta, J. D. (2020). Transboundary Animal Diseases (TADs) affecting domestic and wild African ungulates: African swine fever, foot and mouth disease, Rift Valley fever (1996–2018). *Research in veterinary science*, *131*, 69-77.

Kurpiers, L.A., Schulte-Herbrüggen, B., Ejotre, I., Reeder, D.M. (2016). *Bushmeat and Emerging Infectious Diseases: Lessons from Africa*. In: Angelici, F. (eds) Problematic Wildlife. Springer, Cham. https://doi.org/10.1007/978-3-319-22246-2_24

Mitchell, P. J. (2018). The constraining role of disease on the spread of domestic mammals in sub-Saharan Africa: A review. *Quaternary International*, *471*, 95-110.

Muehlenbein, M. P. (2017). Primates on display: potential disease consequences beyond bushmeat. *American Journal of Physical Anthropology*, *162*, 32-43.

Ohiolei, J. A., Li, L., Ebhodaghe, F., Yan, H. B., Isaac, C., Bo, X. W., ... & Jia, W. Z. (2020). Prevalence and distribution of Echinococcus spp. in wild and domestic animals across Africa: A systematic review and meta‐analysis. *Transboundary and Emerging Diseases*, *67*(6), 2345-2364.

**3. Immunocompetence of the patient**

Because of physiological, metabolic and immunological immaturities, infants and children are more at risk of infection than adolescents and adults (Brodin and Davis, 2017 and references therein; Chitre *et al*., 2024). Additionally, bodies weakened by infections are more likely to suffer from secondary infections (e.g., Ripa *et al*., 2021). Therefore, we have ranked children as highest risk, attributing five points if the zootherapy aims at healing a physical sickness, and four points if it is preventive or aimed at psychological treatments (e.g., ‘against nightmares’). Given that pregnancy impacts the immune system, with a lower adaptive immunity (e.g., decreased number and function of T-cells), but a somewhat stronger innate immunity (e.g., increased number of monocytes, dendritic cells, neutrophils and α-Defensins), we ranked pregnant people as medium risk (Kraus *et al.,* 2011; Pazos *et al*., 2012). Adults were classified as lower risk (one point for preventive or psychological treatments, two points for treatments aimed at physical illnesses).

**References**

Brodin, P., & Davis, M. M. (2017). Human immune system variation. *Nature reviews immunology*, *17*(1), 21-29.

Chitre, S. D., Crews, C. M., Tessema, M. T., Plėštytė-Būtienė, I., Coffee, M., & Richardson, E. T. (2024). The impact of anthropogenic climate change on pediatric viral diseases. *Pediatric research*, *95*(2), 496-507.

Kraus, T. A., Engel, S. M., Sperling, R. S., Kellerman, L., Lo, Y., Wallenstein, S., ... & Moran, T. M. (2011). Characterizing the pregnancy immune phenotype: results of the viral immunity and pregnancy (VIP) study. *Journal of clinical immunology*, *32*, 300-311.

Pazos, M., Sperling, R. S., Moran, T. M., & Kraus, T. A. (2012). The influence of pregnancy on systemic immunity. *Immunologic research*, *54*, 254-261.

Ripa, M., Galli, L., Poli, A., Oltolini, C., Spagnuolo, V., Mastrangelo, A., ... & Vinci, C. (2021). Secondary infections in patients hospitalized with COVID-19: incidence and predictive factors. *Clinical Microbiology and Infection*, *27*(3), 451-457.

**4. Treatment type**

The type of treatment can alter the likelihood of pathogens being transmitted from animal products to humans, for instance considering the nature of the use (e.g., topical application, ingestion), the nature of within-host barriers (e.g., healthy versus damaged epithelial barriers) or the preparation of the animal product (e.g., raw, altered through drying, cooked) (Mataragas *et al*., 2008; Plowright *et al*., 2017). As these three factors were usually reported together (e.g., sun-dried and applied to the wound), we grouped them in our analysis under the umbrella term ‘treatment type.’

We ranked the injection or topical application of raw animal products on wounds as highest risk (five points) due to the high likelihood of pathogens overcoming damaged physical barriers (Coates *et al*., 2018). We ranked the topical application of altered animal products (e.g., in alcohol, powdered or dried) on a wound as high risk (four points), because of the possibility that pathogens were not fully destroyed by the alteration process; as well as the spraying, pouring, inhalation, ingestion, or application on a mucosa of raw animal products, as these are more permeable, but not damaged, physical barriers (Plowright *et al.,* 2017). We ranked the ingestion or topical application on mucosa of altered animal products as medium risk, as well as the topical application of altered/non-altered products on the skin, as undamaged skin represents a strong barrier to pathogen entry (Coates *et al*., 2018). Finally, we ranked the inhalation of altered products and the ingestion of cooked products as lower risk (two points) and lowest risk (one point), respectively, as the likelihood of pathogens reaching from altered animal tissue through physical barriers of the respiratory tract appears low, and the uptake if pathogens from cooked animal tissue through physical barriers of the digestive system appears even lower (Mataragas *et al*., 2008). While tissue type (e.g., fur, blood) could be determined for all included studies (sometimes in their supplementary material), the treatment type could not be determined for at least one of the zootherapeutic practice recorded in 26/53 studies, leading to the attribution of the mean treatment score value to 42% of the practices in our dataset (*i.e*., 1.45, n = 1032).

**References**

Coates, M., Blanchard, S., & MacLeod, A. S. (2018). Innate antimicrobial immunity in the skin: A protective barrier against bacteria, viruses, and fungi. *PLoS pathogens*, *14*(12), e1007353.

Mataragas, M., Skandamis, P. N., & Drosinos, E. H. (2008). Risk profiles of pork and poultry meat and risk ratings of various pathogen/product combinations. *International journal of food microbiology*, *126*(1-2), 1-12.

Plowright, R. K., Parrish, C. R., McCallum, H., Hudson, P. J., Ko, A. I., Graham, A. L., & Lloyd-Smith, J. O. (2017). Pathways to zoonotic spillover. *Nature Reviews Microbiology*, *15*(8), 502-510.

**5. Tissue type**

A great diversity of pathogens can be found at high concentrations in the blood and internal organs of both wild and domestic African animals, ranging from *Babesia* spp protozoans to filoviruses (e.g., Berggoetz *et al*., 2019; Munyeku-Bazitama *et al.*, 2024), which is why we attributed the highest risk score to blood and internal organs, as well as whole animals. Similarly, many pathogens are found in secretions and excreta, such as lyssaviruses in bat saliva (Markotter et al., 2020), or gastrointestinal parasites in dog faeces (Idrissi *et al*., 2022), which is why we ranked this as high risk. Bones can host several bacteria, such as *Staphylococcus* spp (Muthukrishnan *et al*., 2019) and *Mycobacterium* spp (Dibaba *et al*., 2019) – which can also be found on the skin and pulmonary tract. Additionally, bone marrow has been involved in long-term infections of *Brucella* spp in mice (Gutiérrez-Jiménez *et al*., 2018) and prions in cattle (Kim *et al*., 2013). This is why we ranked bones as medium risk. Several ectoparasites, bacteria and fungi can be found on fur and feathers of animals worldwide, such as *Criptococcus* spp and *Blastomyces dermatoditis* (Seyedmousavi *et al*., 2015), however, we find these to be of lower risk, partly because of the lower diversity of pathogens, and partly because of the absence of vascularisation within these tissues, which prevents systemic uptake. Finally, we ranked by-products such as honey and butter, as well as scales and horns, as lowest-risk, as these are not vascularised and offer a lower colonisation potential to ectoparasites compared to fur and feathers.

**References**

Berggoetz, M., Schmid, M., Ston, D., Wyss, V., Chevillon, C., Pretorius, A. M., & Gern, L. (2014). Tick-borne pathogens in the blood of wild and domestic ungulates in South Africa: interplay of game and livestock. *Ticks and tick-borne diseases*, *5*(2), 166-175.

Dibaba, A. B., Kriek, N. P., & Thoen, C. O. (Eds.). (2019). *Tuberculosis in animals: an African perspective*. Springer, eBook.

Gutiérrez-Jiménez, C., Hysenaj, L., Alfaro-Alarcón, A., Mora-Cartín, R., Arce-Gorvel, V., Moreno, E., ... & Barquero-Calvo, E. (2018). Persistence of Brucella abortus in the bone marrow of infected mice. *Journal of Immunology Research*, *2018*(1), 5370414.

Idrissi, H., Khatat, S. E. H., Duchateau, L., Kachani, M., Daminet, S., El Asatey, S., ... & Sahibi, H. (2022). Prevalence, risk factors and zoonotic potential of intestinal parasites in dogs from four locations in Morocco. *Veterinary Parasitology: Regional Studies and Reports*, *34*, 100775.

Kim, Y., Nowzari, H., & Rich, S. K. (2013). Risk of prion disease transmission through bovine‐derived bone substitutes: a systematic review. *Clinical implant dentistry and related research*, *15*(5), 645-653.

Markotter, W., Coertse, J., De Vries, L., Geldenhuys, M., & Mortlock, M. (2020). Bat‐borne viruses in Africa: a critical review. *Journal of zoology*, *311*(2), 77-98.

Munyeku-Bazitama, Y., Edidi-Atani, F., & Takada, A. (2024). Non-Ebola Filoviruses: Potential Threats to Global Health Security. *Viruses*, *16*(8), 1179.

Muthukrishnan, G., Masters, E. A., Daiss, J. L., & Schwarz, E. M. (2019). Mechanisms of immune evasion and bone tissue colonization that make Staphylococcus aureus the primary pathogen in osteomyelitis. *Current osteoporosis reports*, *17*, 395-404.

Seyedmousavi, S., Guillot, J., Tolooe, A., Verweij, P. E., & De Hoog, G. S. (2015). Neglected fungal zoonoses: hidden threats to man and animals. *Clinical Microbiology and Infection*, *21*(5), 416-425.
